# Biomagnification and body distribution of ivermectin in dung beetles

**DOI:** 10.1038/s41598-020-66063-0

**Published:** 2020-06-03

**Authors:** José R. Verdú, Vieyle Cortez, Antonio J. Ortiz, Jean-Pierre Lumaret, Jorge M. Lobo, Francisco Sánchez-Piñero

**Affiliations:** 1 I.U.I. CIBIO, Universidad de Alicante, Alicante, E-03690 Spain; 2 Departamento de Química Inorgánica y Química Orgánica, Universidad de Jaén, Campus Las Lagunillas, Jaén, E-23071 Spain; 3 Université Paul Valéry Montpellier 3, Univ. Montpellier, EPHE, CNRS, IRD, CEFE UMR 5175, F34000. Université Paul-Valéry Laboratoire Zoogéographie, route de Mende, 34199 Montpellier, cedex 5 France; 4 Museo Nacional de Ciencias Naturales-CSIC, Departamento de Biogeografía y Cambio Global. José Abascal 2, Madrid, E-28006 Spain; 5 Departamento de Zoología, Universidad de Granada, Granada, E-18071 Spain

**Keywords:** Agroecology, Entomology

## Abstract

A terrestrial test system to investigate the biomagnification potential and tissue-specific distribution of ivermectin, a widely used parasiticide, in the non-target dung beetle *Thorectes lusitanicus* (Jekel) was developed and validated. Biomagnification kinetics of ivermectin in *T. lusitanicus* was investigated by following uptake, elimination, and distribution of the compound in dung beetles feeding on contaminated faeces. Results showed that ivermectin was biomagnified in adults of *T. lusitanicus* when exposed to non-lethal doses via food uptake. Ivermectin was quickly transferred from the gut to the haemolymph, generating a biomagnification factor (BMF_k_) three times higher in the haemolymph than in the gut after an uptake period of 12 days. The fat body appeared to exert a major role on the biomagnification of ivermectin in the insect body, showing a BMF_k_ 1.6 times higher than in the haemolymph. The results of this study highlight that the biomagnification of ivermectin should be investigated from a global dung-based food web perspective and that the use of these antiparasitic substances should be monitored and controlled on a precautionary basis. Thus, we suggest that an additional effort be made in the development of standardised regulatory recommendations to guide biomagnification studies in terrestrial organisms, but also that it is necessary to adapt existing methods to assess the effects of such veterinary medical products.

## Introduction

Macrocyclic lactones are characterised by a 16-membered cyclic pharmacophore coupled with spiroketal and benzofuran fragments^1^. Ivermectin, a macrocyclic lactone derived from fermentation products of *Streptomyces avermitilis*, is commonly used in veterinary medicine to treat livestock diseases caused by gastrointestinal worms, lung worms and ectoparasites, such as mites and blood-feeding insects^2^. After ivermectin is administered to cattle, its metabolic break down into monosaccharide (22,23-dihydroavermectin B1 monosaccharide) and the aglycon of ivermectin (22,23-dihydroavermectin B1 aglycon) is generally low^3,4^, and between 62–98% of the ivermectin administered may be excreted unaltered almost exclusively in faeces^5,6^ as an unchanged residue retaining its insecticidal activity^7^. The concentration and elimination of ivermectin residues found in excreted dung varies according to the supply method, dosage and diet. When cows were injected with a subcutaneous injection, after 28 days, ivermectin is still detectable in faeces at a concentration of 80 μg kg^–1^ dry weight (~ 10 μg kg^–1^ fresh weight)^8^, and even after 180 days and 13 months, ivermectin residues can be detected in the dung and the soil beneath cattle dung^9,10^. Due to its action on both glutamate-gated chloride (GluCl) and γ-aminobutyric acid (GABA) ion channels, ivermectin potentially affects both target and non-target Ecdysozoan species^11^, and dung beetles are particularly sensitive at both sub-lethal and pre-lethal levels^12–17^. From a functional viewpoint, dung beetles are one of the most important groups using and recycling dung pats in terms of diversity, abundance, and biomass^18^. For this reason, several studies on the ecotoxicology of ivermectin published in the last few decades have focused on the negative effects of ivermectin on this group of insects^19^. Although these studies are relatively numerous, no data exist about the toxicokinetic response to ivermectin in dung beetles during the uptake and elimination phases.

Ecotoxicological assessments based on bioaccumulation, bioconcentration and biomagnification tests are essential for the determination of the environmental risk of chemical compounds as part of the European Commission’s Registration, Evaluation, Authorization, and Restriction of Chemicals programme (REACH, Annex XIII; see https://ec.europa.eu/growth/sectors/chemicals/reach_en). The only established test for bioaccumulation assessment on terrestrial animals was conducted with oligochaetes (Lumbricidae and Enchytraeidae) according to OECD guidance document no. 317^20^. In this test, terrestrial oligochaetes were exposed to contaminated soil via several uptake routes, including water, dermal contact, and ingestion of contaminated soil, yielding a bioaccumulation factor as an endpoint. Biomagnification should be regarded as a particular case of bioaccumulation in which the chemical concentration in an organism is due to dietary absorption^21^. Only a test system to investigate the biomagnification of an organic chemical (hexachlorobenzene) in the terrestrial isopod *Porcellio scaber* Latreille has been recently developed^22^.

A terrestrial test system that considers the dietary pathway from livestock faeces to dung beetles is presented here for the first time. Using the dung beetle *Thorectes lusitanicus* (Jekel) (Geotrupidae) as test species, the gut, haemolymph, fat body and excreta belonging to specimens of this species were examined at different times after ivermectin uptake to analyse its incorporation into the target organs, providing a first overview and point of reference about the pharmacokinetic behaviour of this veterinary medical product in dung beetles.

## Results

### Validity of the performed toxicological test

The behaviour of the studied beetles under laboratory conditions was normal, and no pre-lethal symptoms or mortality were observed in anyone of the individuals treated with the 10.0 μg kg^−1^ dose of ivermectin selected for the experiment (see Table 1). Furthermore, no significant differences were observed in body weight (fresh weight, fw), lipid content and food uptake between control and treated individuals (Table 1). Thus, according to our results, the feeding rate remained stable and without any negative impact over the obtained parameters along the entire study period (24 days).

**Table 1:** Feeding rate, body parameters and survival of *Thorectes lusitanicus* during the entire test period of 24 days.

	Feeding rate (g day^–1^ g^–1^)	Body weight (g)	Lipid content (% body mass)	Survival (%)	Sample size (*n*)
Treated	0.22 ± 0.08	0.68 ± 0.09	6.2 ± 2.3	100	18
Control	0.24 ± 0.03	0.60 ± 0.05	5.1 ± 2.4	100	18
*t* (*P*)	0.79 (0.43)	1.38 (0.19)	1.11 (0.29)		

Values are presented as the mean ± standard deviation. The treated and control values were compared using 1-tailed unpaired *t* tests. There were no significant differences.

### Biomagnification of ivermectin

Using the LC/ESI^+^–MS/MS system, the measured ivermectin concentration in the spiked dung (11.4 ± 2.4 μg kg^−1^: mean ± sd; fw) was similar to the target concentration (10.0 μg kg^−1^), thus indicating a well-adjusted and standardised feeding rate of ivermectin in the beetles during the uptake phase.

**Figure 1:**
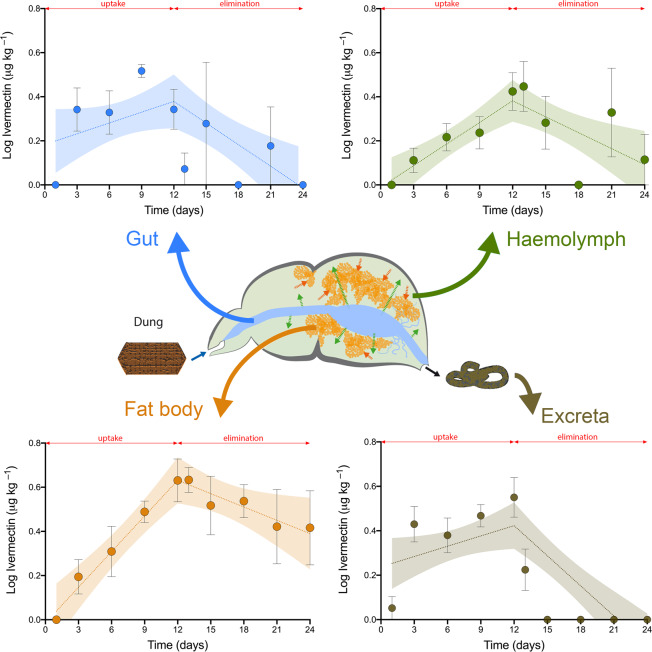
Toxicokinetic curves for ivermectin concentrations in the different biological matrices of *Thorectes lusitanicus*. The red arrow indicates the time that separates the phases of uptake and elimination. Shaded areas represent the 95% confidence intervals of each model. Bars represent ± sem, *n* = 10 for each time period during the uptake phase; for the elimination phase, *n* varied according to the quantity necessary for sample preparation in the extraction protocol (gut: *n* = 3; haemolymph: *n* = 6; fat body: *n* = 6; excreta: *n* = 5).

**Figure 2:**
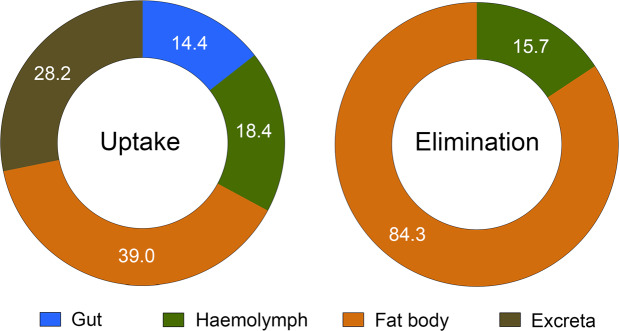
Proportional distribution factors (DF) for ivermectin in different biological matrices of *Thorectes lusitanicus* sampled at day 12 (uptake) and day 24 (elimination). The DF values for each of the four biological matrices are presented as percentages.

From a kinetic viewpoint, the variations in the concentration of ivermectin in the gut, haemolymph, fat body and excreta of *T. lusitanicus* during the complete 24-d biomagnification study period are presented in Fig. 1. During the uptake phase, ivermectin concentration increased gradually in all the examined biological matrices, including the excreta, and steady-state levels were seemingly not reached after the 12 days of the uptake phase. After 3 days of eating, ivermectin was detected in all the analysed biological matrices of *T. lusitanicus* (Fig. 1). After 6 days, the gut (1.50 ± 0.50 μg kg^−1^), excreta (1.75 ± 0.55 μg kg^−1^) and fat body (1.47 ± 0.55 μg kg^−1^) seemed to show higher ivermectin concentrations than haemolymph (0.80 ± 0.25 μg kg^−1^). At the end of the uptake phase, the ivermectin concentration showed a notable increase in the excreta (3.08 ± 1.03 μg kg^−1^), fat body (4.25 ± 1.34 μg kg^−1^) and haemolymph (2.01 ± 0.67 μg kg^−1^) in comparison with that of the gut, which almost remained constant (1.57 ± 0.52 μg kg^−1^). This temporal increase of ivermectin concentrations in the haemolymph and fat body is reflected by their uptake rate values being the highest ones obtained (k_s_; see Table 2). Furthermore, distribution factors (DF) suggested a clear transference of ivermectin from the gut to the haemolymph and the fat body, thus indicating a net increase of the accumulated ivermectin (DF_gut_ = 14.4%; DF_haemolymph_ = 18.4%; and DF_fat body_ = 39.0%). The DF_excreta_ after 12 days of uptake was 28.2% (Fig. 2), suggesting a metabolic effort to eliminate ivermectin.

**Table 2:** Kinetic parameters and biomagnification of ivermectin in the different biological matrices of *Thorectes lusitanicus*.

	k_s_ (95% CI)	k_e_ (95% CI)	BMF_k_ (95% CI)^a^	*R*^*2*^ (%)
Gut	0.016 (0.007–0.025)	0.033 (0.024–0.042)	0.50 (0.21–0.79)	17.5
Haemolymph	0.033 (0.026–0.040)	0.024 (0.016–0.032)	1.63 (0.93–1.70)	23.5
Fat body	0.054 (0.046–0.062)	0.020 (0.011–0.029)	2.68 (2.57–2.79)	37.5
Excreta	0.015 (0.007–0.023)	0.045 (0.037–0.053)	0.34 (0.18–0.50)	31.7

Parameters are the biomagnification factor (BMF_k_), the uptake rate constant (k_s_), and the elimination rate constant (k_e_). Values are presented as the mean and the 95% confidence interval.

^a^Biomagnification threshold is equal to 1.

The elimination phase is characterised by clear differences in the depuration rate between the four analysed biological matrices of *T. lusitanicus* (Fig. 1). Ivermectin was detected after 9 days of elimination in the gut and hemolymph, although it was not detected in the previous analyses (6 days of elimination), probably due to the physiological heterogeneity among individuals used in the study. After 12 days of drug depuration, ivermectin was still detected in the haemolymph and fat body (0.55 ± 0.24 μg kg^−1^ and 2.94 ± 1.20 μg kg^−1^, respectively), showing significant DF values (DF_haemolymph_ = 15.7%; DF_fat body_ = 84.3%) (Figs. 1 and 2). The differences in these kinetic performances during the elimination phase are corroborated by the obtained k_e_ values (Table 2). Accordingly, BMF_k_ values clearly indicate that ivermectin was accumulated in the fat body and, to a lesser extent, in the haemolymph of *T. lusitanicus* 24 days after consumption of dung with ivermectin and after 12 days of drug depuration (Table 2).

## Discussion

Studies on the effects of veterinary medical products on non-target organisms with emphasis on dung feeding invertebrates are very numerous^19^. However, no regulatory guidelines, other than the OECD earthworm bioaccumulation test^20^, directly address the potential bioaccumulation of these compounds in other terrestrial organisms, and very few studies have examined the biomagnification of organic chemicals in terrestrial arthropods^22^. In the case of ivermectin, only a few studies addressed its bioaccumulation in aquatic invertebrates, such as the sediment-dwelling worm *Lumbriculus variegatus* Müller (Oligochaeta)^23^, the zooplanktonic microcrustacean *Ceriodaphnia dubia* Richard (Branchiopoda), the amphipod *Hyalella* sp. (Malacostraca), and the apple snail *Pomacea* sp. (Gastropoda)^24^. The present study is thus the first to analyse the bioaccumulation of an organic chemical, such as ivermectin, in dung beetles.

Dung beetles are undoubtedly one of the most abundant and specialised groups within the beneficial dung insect community^18^ and are therefore at special risk due to the contamination of dung by veterinary medical products such as ivermectin^14^. This study clearly shows that ivermectin is biomagnified in adults of *T. lusitanicus* when exposed to non-lethal doses via food uptake. From a pharmacokinetic viewpoint, our results suggest that ingested ivermectin was quickly transferred from the gut to the haemolymph, generating a biomagnification factor (BMF_k_) three times higher in the haemolymph than in the gut after 12 days of uptake (see Table 2). This biomagnification in the haemolymph could explain the acute toxicity of ivermectin on adult dung beetles, yielding sub-lethal effects that seriously affect sensorial and locomotor capacities even at very low doses^13,15^. The mechanisms involved in the transfer of macrocyclic lactones through the digestive system of insects are unknown, but other insecticides such as organochlorines, organophosphates, pyrethroids, and neonicotinoids that are characterised, as ivermectin is, by their high lipophilicity, may easily move through the digestive system to their site of action^25^. Our results suggest that ivermectin moves rapidly through the gut and that also accumulates in the haemocoel, subsequently reaching different organs in direct contact with the haemolymph, such as fat body tissues. Exhibiting a typical pharmacokinetic behaviour, the fat body appears to exert a major role on the biomagnification of ivermectin in the insect body, showing a BMF_k_ 1.6 times higher than that of the haemolymph (Table 2). This result is consistent with the lipophilicity of ivermectin and is in agreement with its behaviour in other fatty tissues, such as the adipose tissue of vertebrates^26^ (see Table S2 in Supplementary Information). In insects, the fat body is an aggregated loose mass of adipocytes irregularly distributed within the haemocoelic space, immersed in haemolymph, and surrounding the gut and the reproductive organs^27^. Fat body plays an essential role in nutrient storage and metabolism, being responsible for the synthesis of the fatty acids and proteins found in the haemolymph^28^. The BMF_k_ value of the fat body supports the idea that the biomagnification of ivermectin in the fat body could cause unsuspected toxicological consequences. On the one hand, the sequestration of ivermectin by adipocytes could decrease the toxicant concentration in the haemolymph, reducing ivermectin availability to other vital tissues and organs where they may generate harmful effects, such as sensorial and reproductive organs or muscular tissues^13,15,29^. On the other hand, biomagnification of ivermectin in the fat body of dung beetles feeding on contaminated dung for extended periods of time could cause a significant increase in body burden. During periods of energy demands, fatty acids stored in the fat body are mobilised for a number of purposes, including the provision of energy to flight muscles, the movement of lipids to the ovaries during vitellogenesis, and the overall maintenance of the metabolic activity of other tissues, especially during periods of starvation^28^. In the dung beetle species *Euoniticellus intermedius* (Reiche), yolk synthesis is rapid, and females need to feed throughout the oviposition period. When present in dung, ivermectin alters the morphology of the ovary and stops vitellogenesis, causing oocyte resorption and a decrease of fecundity^16^. In vertebrates, ivermectin also accumulates in fatty tissues. When ivermectin is administered to laying hens, it is preferentially deposited in the egg yolk, where residue levels were higher than those found in other tissues^30,31^.

Further studies analysing different species across the trophic chain are needed to explore the existence of a biomagnification process at the level of the dung-based food web. The results of this study highlight that the bioaccumulation of ivermectin must be investigated from a global dung-based food web perspective, and also that the use of the veterinary medical products must be monitored and controlled following a precautionary principle.

Finally, we suggest that an additional effort should also be made in the development of standardised regulatory recommendations to guide biomagnification studies in terrestrial organisms, and to allow for appropriate regulatory actions for environmental health and the protection of ecosystem services. In addition, we suggest that existing methods for assessing the effects of these veterinary medical products should be improved. The present study provides useful information to achieve these objectives and to elucidate the mechanisms involved in the biomagnification of ivermectin in dung beetles.

## Materials and methods

### Test organism

The dung beetle *T. lusitanicus* was selected as test species. This species has been successfully used in other physiological studies^32,33^ due to its easy rearing and the high volume of haemolymph in its body. Specimens of *T. lusitanicus* were used to study ivermectin biomagnification patterns from cattle dung into the gut, haemolymph, fat body and excreta (biological matrices) to gain insight about the pharmacokinetic behaviour of ivermectin in dung beetles.

In November 2016, individuals of *T. lusitanicus* were collected at La Sauceda, an ivermectin-free site in the Los Alcornocales Natural Park (Cádiz), in Southern Spain (36°31′54″N, 5°34′29″W). We used pitfall traps baited with cow dung to capture live beetles. The dung beetles were maintained in aerated plastic containers (80 × 30 × 50 cm) at 18 °C with 65% relative humidity (RH) and a photoperiod of 14:10 h (light:dark). The substrate was moss and dead fallen leaves of *Quercus* to prevent beetle stress.

In order to standardize the physiological condition of the beetles, only mature specimens were selected according to external age-grading methods (e.g., abrasion of the fore tibiae in conjunction with cuticle hardness of the pronotum and elytra, which makes it possible to sort out the individuals of approximately the same age^34^). In addition, we used a 1:1 sex ratio in each experiment. This work conforms to the Spanish legal requirements including those relating to conservation and welfare.

### Biological matrices

To determine the accumulation of ivermectin in dung beetles, four different matrices (gut, haemolymph, fat body, and excreta) were chosen. Ivermectin concentrations in the gut and excreta were analysed because they indicate the amount of ivermectin ingested and excreted, respectively. Haemolymph has been selected since it is the fluid in direct contact with all tissues and organs, and is responsible for the distribution of a large number of biological constituents to the cells (amino acids, proteins, lipids, and carbohydrates, among others). Fat body was selected because it constitutes the main tissue for nutrient storage, intermediary metabolism and regulation being the insect equivalent to the adipose tissue and liver in vertebrates^28^.

### Application of the test substance and preparation of experimental diets

For this toxicological test, a concentration of 10 μg kg^−1^ (fresh weight) of ivermectin (≥90% B1a; ≤5% B1b percent purity, Sigma Aldrich Co., St. Louis, USA) in dung and an untreated dung control were used. The ivermectin concentration used was chosen because it is closely related to the toxicity threshold corresponding to the inhibition of antennal response in dung beetles (IC_50_ = 8.16 μg kg^−1^), as previously reported by Verdú et al.^15^. This concentration was also selected because it corresponds to the OECD guidelines, which suggest that the chosen concentration should be low enough to ensure that it does not affect insect behaviour while being high enough to allow its quantification throughout the uptake and elimination phases^20^. Dung treatments were prepared using the procedure developed by Verdú et al.^13^, in which a single concentration of ivermectin was spiked into fresh cow dung. The solution was made by dissolving ivermectin in absolute ethanol (Sigma-Aldrich Co.), subsequently added to 2 kg of fresh dung, and mixed for 2 h using a dough mixer machine. The concentration of ivermectin in the dung was determined after spiking to verify its homogenous distribution in the dung. The untreated dung control was processed in an identical manner but without the addition of ivermectin. Dung treatments were exposed to air for 4 h to remove residual ethanol and then placed in sealed plastic buckets to prevent desiccation during storage at 5 °C until use.

### Toxicological test design

According to the OECD standard technical guide^20^, we implemented a continuous 12-day uptake phase. Dung beetles (n = 18 individuals per treatment) were sampled at 1, 3, 6, 9 and 12 days after the first addition of dung with ivermectin. After this 12-day uptake phase, the dung beetles were transferred to clean test boxes for the subsequent 12-day elimination phase also sampled at 1, 3, 6, 9 and 12 days. The experiments are described in the sections below.

### Experimental procedure

During the continuous 12-day uptake phase, beetles were provided daily with 2 g of dung containing a concentration of 10 μg kg^−1^ (fresh weight) of ivermectin. After this 12-day uptake phase, the same individuals were provided with the same quantity (2 g) of ivermectin-free dung and were sampled again during another 12-days period (at 1, 3, 6, 9 and 12 days after the end of the first feeding phase). At each sampling time, food remains were removed, and their dry weight recorded to estimate the feeding rate per individual, which was standardised according to the fresh body weight of each individual (g day^−1^ g^−1^). During all the experimental period, dung beetles were maintained individually in plastic containers (60 × 40 × 40 cm) with moist sterile paper as a substrate at 18 °C (a temperature similar to the optimal temperature experienced in the field) within a climate-controlled chamber at 65% relative humidity (RH) and a photoperiod of 14:10 h (light:dark).

### Validity of the test

In addition, to control the potential negative effects of ivermectin on beetle behaviour (pre-lethal symptoms), feeding rate, body weight, lipid content, and survival, 36 individuals were randomly assigned to two treatments: i) a control treatment (n = 18 individuals), in which beetles were fed with ivermectin-free dung during the whole 24 days experiment; and ii) an ivermectin treatment (n = 18 individuals), in which beetles were fed during 12 days (uptake phase) with spiked dung (containing ivermectin), followed by 12 days of feeding on ivermectin-free dung (elimination phase). Food consumption for each individual was monitored daily by placing 2 g of dung on a plastic dish (4 mm diameter and 13 mm height) to allow measurement of the quantity of dung ingested while avoiding water loss due to absorption by the substrate.

### Sample preparation

To assess the biomagnification patterns and pharmacokinetic behaviour of ivermectin released from cattle dung, we extracted ivermectin from four biological matrices (gut, haemolymph, fat body and excreta) of *T. lusitanicus*. At the end of each of the ten sampling times (five during the uptake phase and five during the elimination phase), the gut, fat body, haemolymph and defecated beetle excreta were obtained from 10 randomly selected individuals. After each individual was anaesthetised with CO_2_, haemolymph samples were collected by puncturing the cuticle on the dorsal side of the pronotum and gently squeezing the beetle as described previously by Verdú *et al*.^32^. Haemolymph collected from each individual was placed into a vial, protected from light and maintained at –25 °C. Next, the beetle was placed on a dissection tray in which distilled water was added. The gut samples were obtained by dissecting from the foregut (stomodeum) to the hindgut (proctodeum) with the help of micro-clamps sealing both ends to avoid possible contamination. After dissection, the external surface was thoroughly washed with distilled water to rinse away any haemolymph that remained on the surface. Based on a previous work with the same test species^32^, to determine the lipid content, all of the fat body tissue was removed, placed on a dry paper towel to remove haemolymph and dried at 28 °C for 24 h to eliminate excess water prior to weighing using an AS 82/220.R2 analytical balance (RADWAG USA L.L.C., North Miami Beach, FL, USA). The samples of fat body and gut were taken from each individual and preserved in 300 µl of absolute ethanol. Before extraction process, the absolute ethanol from fat body and gut samples was evaporated with a gentle stream of nitrogen until dryness at room temperature. The excreta samples collected after defecation from each individual were dried and homogenised with liquid nitrogen and were then placed into plastic vials. To reach the minimum amount of material necessary to perform the analyses (0.3 ± 0.01 g for each sample) it was necessary to homogenise the samples from two individuals for each time period and biological matrix, with the exception of the gut on the third day of the uptake phase in which only a sample from a single individual was necessary. All samples were stored at −85 °C in a freezer (SANYO Electric Co. Ltd, Japan) until further analysis.

### Ivermectin extraction

After the samples were collected, the clean up and extraction protocol was based on the optimised method described by Ortiz *et al*.^35^. Briefly, 0.3 ± 0.01 g of each sample (gut, haemolymph, fat body or excreta) was weighed in a 10 ml centrifuge tube, and 1 ml of a solution of acetonitrile:water (60:40) and 200 µl of the internal standard (IS) working solution (Abamectin, 10 ng ml^−1^) were added. The mixture was vortexed (REAX Control, Heidolph, Kelheim, Germany) during 1 min. After vortex mixing, the sample was sonicated using an ultrasonic homogenizer (SONOPLUS Ultrasonic Homogenizers HD 3200, 200 W, 20 KHz) at 40% power for 5 min. The tubes were centrifuged (BL-II, JP Selecta, Barcelona, Spain) at 5000 rpm for 10 min. The supernatant extracts were carefully removed, filtered (PTFE syringe filter 0.20 µm, Millex® Millipore Ibérica, Madrid, Spain) and transferred to a clean tube where it were evaporated to dryness at 45 °C under a stream of ultrahigh-purity N_2_. After reconstitution with 5 ml of pure water, the resulting solution was ultrasonicated using a water bath (JP Selecta, Barcelona, Spain) for 2 min and was filtered through a 0.45 µm PTFE syringe filter (Millex^®^ Millipore Ibérica, Madrid, Spain) to remove any precipitate and to prevent the suspended particles from reaching the continuous unit.

The pre-concentration and clean-up of samples were performed using continuous solid phase extraction (SPE). Before pre-concentration of each sample, the SPE column was conditioned passing through the sorbent 1 ml of acetonitrile, 1 ml of methanol and 5 ml of purified water. The continuous SPE technique used was assembled from a Minipuls-3 peristaltic pump (Gilson, Middleton, WI, USA) fitted with polyvinyl chloride (PVC) pumping tubes, two Rheodyne 5041 injection valves (Cotati, CA, USA), PTFE tubing (3 mm I.D.) and a laboratory-prepared sorption column containing 80 mg Oasis-HLB^®^ sorbent (5 cm × 3 mm i.d.). In the concentrating step, 5 ml of the reconstituted aqueous sample was passed at 4 ml min^−1^ through the sorbent column, thus the ivermectin and IS were adsorbed and the sample matrix was sent to waste. The analytes were eluted with 300 µl of acetonitrile into a glass vial and stored at −20 °C until analysis.

### Reagents and chemicals

Standard grade ivermectin (≥90% B1a ≤5% B1b purity), and abamectin (≥98.7% purity) were purchased from Sigma-Aldrich (MO, USA) and stored at −20 °C. Abamectin, a precursor of ivermectin, differs from ivermectin in that it has a double bond at the C_22–23_ position^34^ and was used as an internal standard (chemical structures and physical chemical properties of ivermectin and abamectin are described in Supplementary Table S1 and Fig. S1). Oasis-HLB^®^ sorbent was obtained from Waters Corporation (Milford, Massachusetts, USA). Ammonium formate and formic acid (analytical reagent grade) were purchased from Merck (Darmstadt, Germany). Millex-LG filter units (hydrophilic, PTFE, pore size = 0.20 and 0.45 μm, diameter = 25 mm, filtration area = 3.9 cm^2^) were obtained from Millipore Ibérica (Madrid, Spain). Water was purified by a Milli-Q purification system (Millipore Ibérica, Madrid, Spain). All other reagents used were purchased from standard suppliers and of analytical grade or higher.

### Stock solutions

The method of standardization was based on the use of abamectin as internal standard, which was added to the samples just before the extraction procedure. Standard solutions were prepared as follows: to prepare the stock solutions, 1.0  ± 0.001 mg of ivermectin and abamectin reference standards were accurately weighed into individual 100 ml volumetric flasks and dissolved using methanol to prepare two stock solutions at a final concentration of 10 μg ml^−1^. Working solutions of ivermectin ranging from 0.1 to 10 ng ml^−1^ were prepared by appropriate dilution of the stock solution with methanol. The method of standardization was based on the use of abamectin as internal standard, which was added to the samples just before the extraction procedure. The internal standard working solution concentration was 10 ng ml^−1^. All of the solutions were stored in a freezer at −20 °C.

### LC/ESI^+^–MS/MS instrumentation and settings

We applied the method proposed by Ortiz *et al*.^35^ for the quantitative determination of ivermectin. Briefly, ivermectin was analysed on an Agilent 1100 HPLC system, which was coupled to an Ion Trap MS analyser (Esquire 6000, Bruker Daltonics, Bremen, Germany) equipped with an electrospray ionization source (ESI). The system was controlled with the Agilent ChemStation (version A.06.01, Agilent Technologies) and Bruker Daltonics Esquire control (version 6.08, Bruker Daltonics) software packages. The data were processed with Data Analysis software (version 3.2, Bruker Daltonics).

A C_18_ Kinetex analytical column (75 mm × 3.0 mm, 3.0 μm, Phenomenex, Torrence, CA, USA) was used and it was operated at 40 °C. The mobile phase solvent A was a solution of 0.1% (v/v) formic acid in water, and solvent B was 0.1% (v/v) formic acid in acetonitrile. The column flow rate was 0.25 ml min^−1^, the injection volume was 1 μl and the gradient elution timetable was as follows: 0–10 min, 50% A-50% B; 10–15 min, 100% B; 15–20 min 50% A-50% B.

The MS/MS conditions were initially optimized by injecting standards at concentration of 1 μg/ml of ivermectin and internal standard solution. The optimal MS/MS sensitivity was obtained using electrospray in positive ionization (ESI^+^) and for quantitative purposes, the instrument was operated in multiple reaction monitoring (MRM) mode, scanning from 50 to 2000 m/z. In positive ion mode, a precursor ion at *m/z* 897 [M + Na]^+^ was observed for ivermectin, similar to results shown for IS at *m/z* 895 [M + Na]^+^. For ivermectin, a major product ion at *m/z* 753.1 [M-144+Na]^+^ was observed an identical adduct ion for abamectin at m/z 751.1. Since the [M-144+Na]^+^ was the product ion with the greatest abundance, it was chosen for quantitation of both compounds.

The method for the quantitative determination of ivermectin in the different biological matrices was validated by linearity of the method, lower limit of detection (LLOD), lower limit of quantification (LLOQ) and percentage of recovery^35^ (see Supplementary Table S2).

### Data analysis and statistics

Following OECD guidelines for the testing of chemicals^20^, if a steady state is not achieved within the uptake phase, the dietary biomagnification factor (BMF_k_) must be determined as the ratio between the uptake (k_s_) and elimination (k_e_) rate constants. For each fraction, the kinetic parameters (k_s_ and k_e_) were calculated in one run by applying a piecewise linear regression model after log transformation of the data from both the uptake and elimination phases, simultaneously. The breakpoint was 12 days coinciding with the time when the beetles have stopped eating dung containing ivermectin. Least squares regression fitting was used to obtain confidence intervals for slope parameters (95% CI) and to quantify the goodness-of-fit of the regression (*R*^2^). To discard any possible negative effects caused by ivermectin, the beetle body weight, lipid content and survival under laboratory conditions were compared between treated and control individuals by means of *t* tests. All data were analysed using the software GraphPad Prism (v8, San Diego, USA).

Distribution factors (DF) were calculated for each tissue fraction to illustrate the differences in ivermectin accumulation rates between the distinct biological matrices of the studied beetles. The proportional distribution of total ivermectin residues in each of the four matrices was thus calculated to evaluate their contribution to the total ivermectin accumulated in all biological matrices at the end of the uptake (day 12) and elimination phases (day 24).

## Supplementary information

Supplementary information S1.

## Data Availability

The datasets analysed during the current study are available from the corresponding author on reasonable request.
