# Supplementary material for: Biomagnification and body distribution of ivermectin in dung beetles
Source: Sci Rep. 2020 Jun 3;10:9073. doi: 10.1038/s41598-020-66063-0 (PMC7270108; doi:10.1038/s41598-020-66063-0)
Supplement: Supplementary file 1 — Supplementary information S1. [file 41598_2020_66063_MOESM1_ESM.docx]

Supplementary information of the paper titled “Biomagnification and body distribution of ivermectin in dung beetles” by José R. Verdú, Vieyle Cortez, Antonio J. Ortiz, Jean-Pierre Lumaret, Jorge M. Lobo, Francisco Sánchez-Piñero

Figure S1. Chemical structure of ivermectin (CAS number: 71827-03-7) and abamectin (CAS number: 71751-41-2)

Table S1. Physical chemical parameters of ivermectin and abamectin.

Table S2. Validation results for the determination of ivermectin in dung beetle sample matrices by LC-ESI^+^-MS/MS.


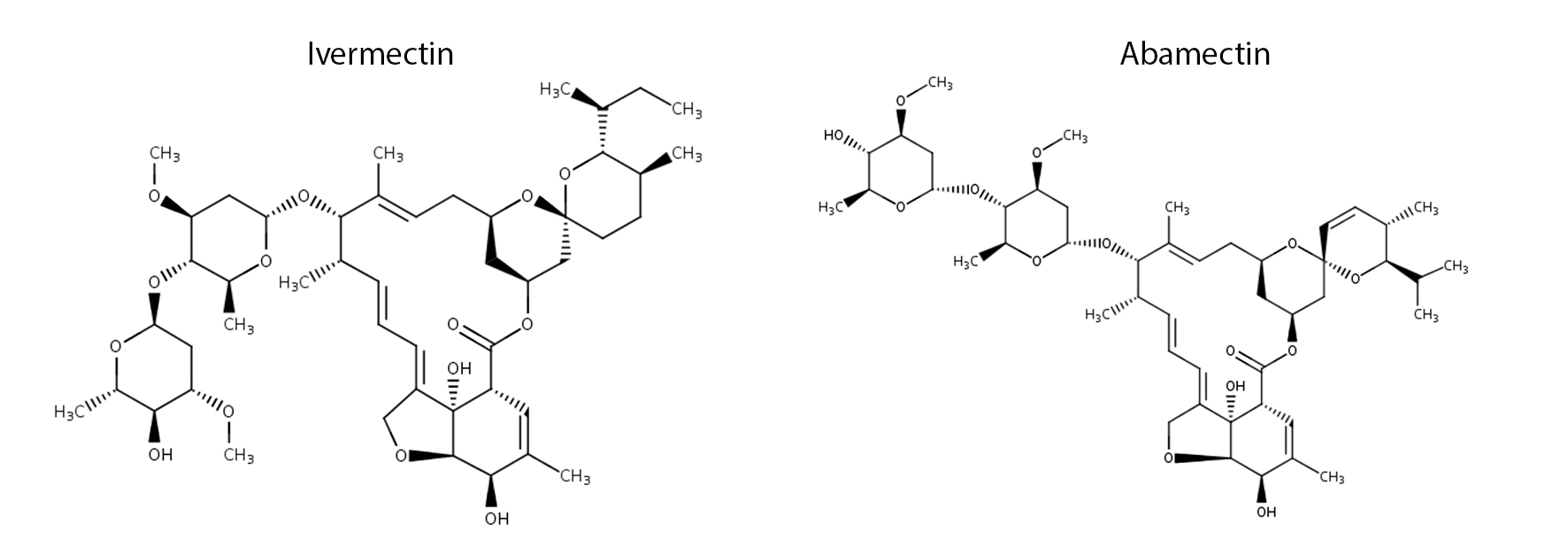


**Figure S1. Chemical structure of ivermectin (CAS number: 71827-03-7) and abamectin (CAS number: 71751-41-2)**

**Table S1. Physical chemical parameters of ivermectin and abamectin (based on references cited below)**

|  | Ivermectin | Abamectin |
| --- | --- | --- |
| Molecular weight | 875.1 g mol^−1^ | 1732.1 g mol^−1^ |
| Kow | 1651 | 9772 |
| Koc | 12660–15700 L kg^–1^ | 5300–15700 L kg^–1^ |
| Aqueous solubility | 4 µg L^–1^ | 7.8 µg L^–1^ |
| Vapour pressure | <1.5 10^–9^ mm Hg | 1.5 10^–9^ mm Hg |
| Photolysis in water | <0.5 days | <0.5 days |
| Soil half-life | 93–240 days | 14–56 days |

**Table S2.** **Validation results for the determination of ivermectin in dung beetle sample matrices by LC-ESI^+^-MS/MS.**

| Parameters | Concentration |  |
| --- | --- | --- |
|  |  | Correlation coefficient (*r*) |
| Calibration curves | 0.1 to 10 ng g^–1^ | 0.988 |
|  | 0.1 to 100 ng g^–1^ | 0.989 |
|  |  | Concentration |
| Lower limit of detection (*LLOD*) | 0.1 to 10 ng g^–1^ | 0.01 ng g^–1^ |
| Lower limit of quantification (*LLOQ*) | 0.1 to 10 ng g^–1^ | 0.1 ng g^–1^ |
|  |  | % Recovery |
| Recovery | 0.6 ng g^–1^ | 91.1 |
|  | 6 ng g^–1^ | 103.2 |

**References**

Halley, B.A.; Nessel, R.J.; Lu, A.Y.H. Environmental Aspects of Ivermectin Usage in Livestock: General Considerations. In: *Ivermectin and Abamectin*; Campbell, W.C., Ed.; Springer-Verlag: New York, 1989; pp. 162-172

Kövecses, J.; Marcogliese, D.J. *Avermectins: Potential Environmental Risks and Impacts on Freshwater Ecosystems in Quebec*. Scientific and Technical Report ST-233E. Environment Canada – Quebec Region, Environmental Conservation, St. Lawrence Centre. 2005, pp. 1-72.
